# Supplementary material for: Deduced Respiratory Scores on COVID-19 Patients Learning from Exertion-Induced Dyspnea
Source: Sensors (Basel). 2023 May 13;23(10):4733. doi: 10.3390/s23104733 (PMC10221378; doi:10.3390/s23104733)
Supplement: Supplementary file 1 [file sensors-23-04733-s001.zip › sensors-2363754-supplementary.pdf]

Supplementary Materials

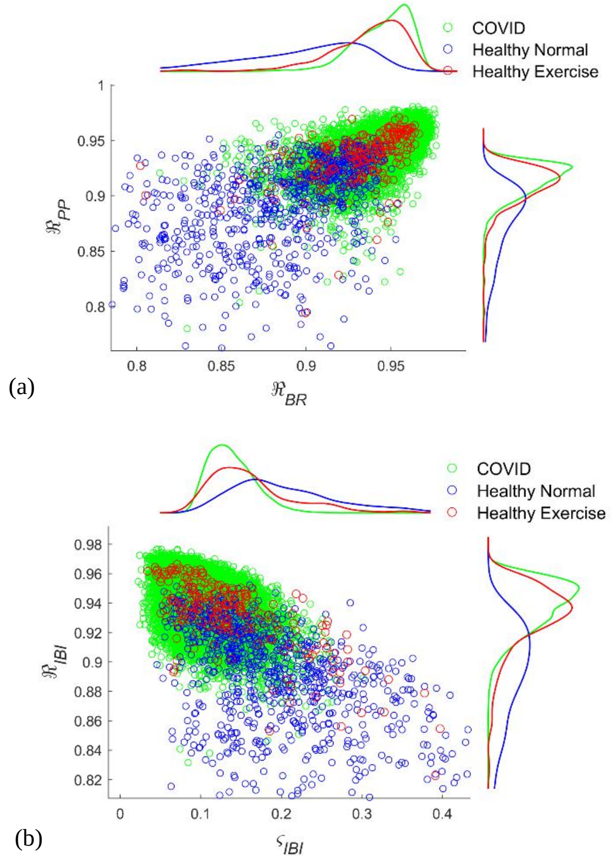

Figure S1. Scatter plots of sample respiratory features from COVID-19 patients (sensor: accelerometer) and healthy subjects in Exp 1 (sensor: accelerometer). Top and right lines are smoothed continuous distribution by Gaussian kernels. **(a)** Autocorrelation of breath rates vs. lung volume ($\Re_{BR}$ vs. $\Re_{PP}$); **(b)** Successive difference vs. autocorrelation of inter-breath intervals (ς_{IBI} vs. $\Re_{IBI}$).

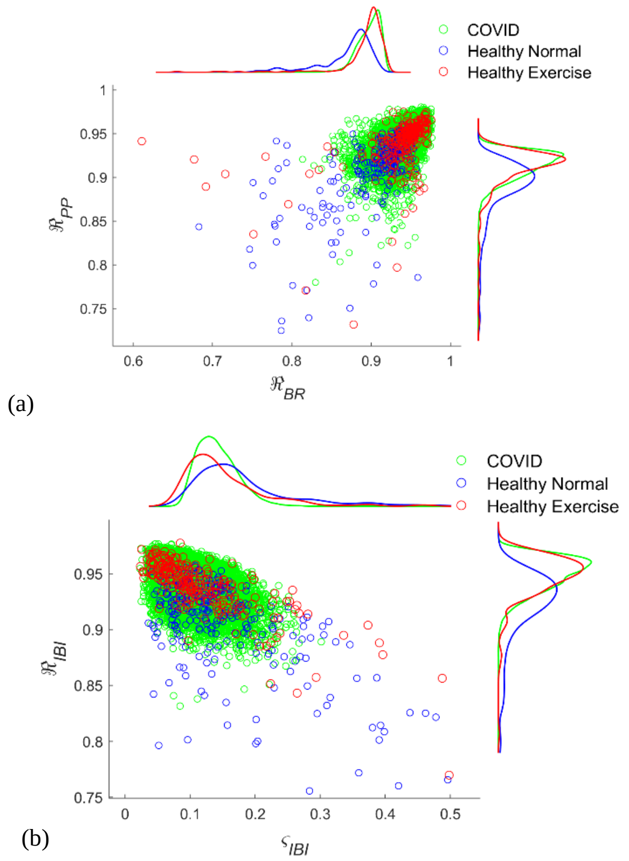

Figure S2. Scatter plots of sample respiratory features from COVID-19 patients (sensor: accelerometer) and healthy subjects in Exp 2 (sensor: NCS). Top and right lines are smoothed continuous distribution by Gaussian kernels. **(a)** Autocorrelation of breath rates vs. lung volume ($\Re_{BR}$ vs. $\Re_{PP}$); **(b)** Successive difference vs. autocorrelation of inter-breath intervals (ς_{IBI} vs. $\Re_{IBI}$).

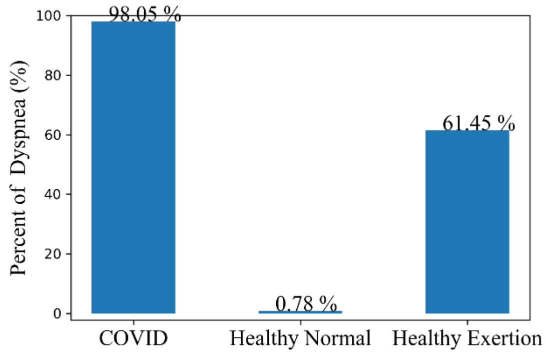

Figure S3. Classification results of dyspnea for COVID-19 patients (sensor: accelerometer) and healthy subjects in Exp 1 (sensor: accelerometer).

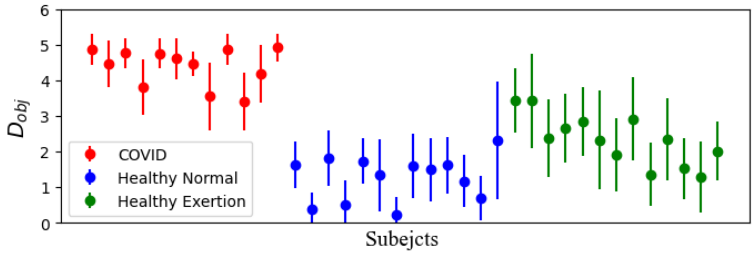

Figure S4. Dyspnea scoring results for COVID-19 patients and healthy subjects in Exp 1 by accelerometer sensing. Average D_{obj} : COVID = 4.39; Healthy Normal = 1.28; Healthy Exertion = 2.35.

Table S1. DEMOGRAPHIC DISTRIBUTION OF COVID-19 PATIENTS.

Case No.	Gender (Male/Female)	Height (ft)	Weight (lb)	BMI
1	F	5' 11"	167	23.3
2	F	5' 0"	123	24.3
3	M	5' 9"	277	40.9
4	M	6' 0"	197	24.3
5	M	5' 8"	198	38.8
6	M	5' 8"	157	24.8
7	M	5' 6"	148	24.3
8	M	5' 11"	201	38.8
9	M	5' 8"	154	24.8
10	M	5' 4"	180	24.3
11	F	5' 8"	254	38.8
12	F	5' 3"	138	24.8

Table S2. DEMOGRAPHIC DISTRIBUTION OF HEALTHY SUBJECTS IN EXP1.

Case No.	Gender (Male/Female)	Height (ft)	Weight (lb)	BMI	Age
1	F	5' 5"	111	18.5	24
2	M	6'	135	18.3	20
3	F	5' 7"	180	28.2	22
4	F	5' 5"	130	21.6	23
5	F	5' 5"	100	16.6	19
6	F	5' 5"	140	23.3	19
7	F	5' 5"	120	20.0	21
8	M	5' 9"	165	24.4	24
9	M	6'	143	19.4	24
10	M	5' 11"	168	23.4	31
11	M	6'	175	23.7	23
12	M	5' 6"	160	25.8	59
13	M	5' 7"	148	23.2	25

TABLE S3. DEMOGRAPHIC DISTRIBUTION OF HEALTHY SUBJECTS IN EXP2.

Case No.	Gender (Male/Female)	Height (ft)	Weight (lb)	BMI	Age
1	M	6'	165	22.4	30
2	M	5' 10"	183	26.3	32
3	M	6' 5"	209	24.8	25
4	M	5' 7"	158	24.7	58
5	M	5' 7"	143	22.4	22
6	F	5' 5"	119	19.8	24
7	M	5' 10"	132	18.9	31
8	F	5' 6"	132	21.3	27
9	F	5' 4"	121	20.8	22
10	M	5' 10"	172	24.7	30
11	M	5' 10"	154	22.1	24
12	F	5' 5"	114	19.0	25
13	F	5' 4"	108	18.5	25
14	F	5' 4"	115	19.7	24
15	M	5' 7"	161	25.2	26
16	M	5' 10"	172	24.7	23
17	F	5' 5"	112	18.6	24
18	F	5' 3"	115	20.4	24
19	F	5' 8"	143	21.7	23
20	M	5' 10"	174	25.0	23
21	M	5' 10"	176	25.3	25
22	M	6'	125	17.0	18
23	F	5' 5"	112	18.6	26

24	F	5' 5"	132	22.0	28
25	F	5' 5"	108	18.0	26
26	F	5'	101	19.7	27
27	F	5' 5"	125	20.8	28
28	F	5' 4"	115	19.7	22
29	M	5' 8"	145	22.0	20
30	F	5 '5"	121	20.1	24
31	F	5 '5"	110	18.3	25
32	F	5' 8"	143	21.7	20

Table S4. REPORTED DYSPNEA AT REST OF COVID-19 PATIENTS AT STUDY ADMISSION.

Case No.	Dyspnea at rest (beginning of recording)
1	YES - significant
2	NO
3	Yes - mild
4	YES - significant
5	NO
6	Yes - moderate
7	Yes - mild
8	YES - significant
9	YES - significant
10	Yes - mild
11	Yes - mild
12	YES - significant
